# Supplementary material for: Young adults who improve performance during dual-task walking show more flexible reallocation of cognitive resources: a mobile brain-body imaging (MoBI) study
Source: Cereb Cortex. 2022 Jun 6;33(6):2573–92. doi: 10.1093/cercor/bhac227 (PMC10016048; doi:10.1093/cercor/bhac227)
Supplement: MoBI_GNG_YA_Supplementary_Material_EP_EGF_bhac227 [file mobi_gng_ya_supplementary_material_ep_egf_bhac227.docx]

**Supplementary Material for**

Young adults who improve performance during dual-task walking show more flexible reallocation of cognitive resources: A Mobile Brain-Body Imaging (MoBI) study.

*Eleni Patelaki^1,2^, John J. Foxe^1^, Kevin A. Mazurek^1,3,4^, Edward G. Freedman^1,*^*

**Addresses:**

^1^ The Frederick J. and Marion A. Schindler Cognitive Neurophysiology Laboratory, The Del Monte Institute for Neuroscience, Department of Neuroscience, University of Rochester School of Medicine and Dentistry, Rochester, New York, USA

^2^ Department of Biomedical Engineering, University of Rochester, Rochester, New York, USA

^3^Department of Physiology and Biomedical Engineering, Mayo Clinic, Rochester, Minnesota, USA

^4^The Well Living Lab, Rochester, Minnesota, USA

***Correspondence:** [ed_freedman@urmc.rochester.edu](mailto:ed_freedman@urmc.rochester.edu)

**Power analysis**

The sample size was calculated a priori. The hypothesis that this study set out to test was that IMPs would differ from nIMPs in terms of dual-task-related neural resource allocation, quantified as walking-minus-sitting ERP difference during the N2, P3 and ERN stages of inhibitory processing. There was no prior data on the difference between IMPs and nIMPs in terms of this dual-task-related ERP amplitudes and, as such, power analysis using an independent-samples t-test (between-subjects variable = Group, independent samples = IMP/nIMP groups) could not be conducted since this effect size was unknown. Consequently, the sample size was estimated by running a power analysis using a paired-samples t-test (within-subjects variable = Motor Load, sitting/walking ERP amplitudes) and doubling the sample size that this analysis yielded. To carry out the power analysis, the G*Power 3.1.9 software was used. The parameters entered into the paired-samples power analysis were the following: tails = 2, error probability α = 0.05, power (1-β) = 0.08, effect size d = 0.86 (see below how this was calculated). A snapshot of the power analysis in G*Power is shown below.

Based on the above, the target sample size was calculated as: 2*13 = 26 subjects.

Effect size calculation:

The effect size was estimated based on data from previous studies. Specifically, in De Sanctis and colleagues (De Sanctis and others 2014), a paired-samples t-test was used to compare sitting and walking (briskly) N2 amplitudes, and the resulting p-value was 0.0001 (page 59 of the De Sanctis and colleagues paper). Similarly, a paired-samples t-test was used to compare sitting and walking (briskly) P3 amplitudes, and the resulting p-value was 0.001 (page 59 of the De Sanctis and colleagues paper). The highest of the two p-values (i.e. 0.001) was kept in order to get a more conservative estimate of the effect size. Of note, the sample size of the De Sanctis and colleagues study is n = 18. The t-value was estimated using Student's t inverse cumulative distribution function in MATLAB 2021a, as follows: tval = tinv(p, df) = tinv(p, n-1) = tinv(0.001, 17) = 3.6458. The effect size was calculated using Cohen's d formula provided by Rosenthal (Rosenthal 1991): d = t/√n = 3.6458/√18 = 0.8593 ≈ 0.86.

## **Pilot Data**

D’ scores

Five (5) participants ages 18-25 (1 female, 4 male) participated in the piloting of the study. All participants reported no diagnosed neurological conditions, no recent head injuries, and normal or corrected-to-normal vision. The d’ scores of the 5 pilot participants on the employed Go-NoGo response inhibition task, during sitting and walking, are listed in Supplementary Table 1. Three (3) out of 5 pilot participants exhibited greater d’ scores, therefore improved response accuracy, during walking compared to sitting. The d’ scores of these 3 participants are highlighted in bold in the table.

Supplementary Table 1. Sitting and walking d’ scores of the five (5) pilot participants. The scores of those who exhibited improved d’ performance during walking compared to sitting are highlighted in bold.

|  | Sitting | Walking |
| --- | --- | --- |
| Participant 1 (P1) | **0.58** | **0.93** |
| Participant 2 (P2) | **3.34** | **3.89** |
| Participant 3 (P3) | 3.84 | 3.30 |
| Participant 4 (P4) | **3.43** | **4.35** |
| Participant 5 (P5) | 4.04 | 3.31 |

Number of trials per condition

Supplementary Table 2 demonstrates the number of trials for each of the four (4) behavioral conditions of the Go-NoGo task (correct rejections, false alarms, hits, misses) and for each of the two (2) motor load conditions (sitting, walking), for each one of the 5 pilot participants. Of note, at the piloting stage, an experimental session consisted of 11 blocks: 1 training block at the beginning, 5 sitting blocks, 5 walking blocks. The only exception was participant 3 (P3) for whom only 2 sitting and 4 walking blocks were recorded, plus training. For the actual cohort, the number of experimental blocks was increased by adding 2 walking blocks (7 in total), 2 sitting blocks (7 in total) and 1 single-task walking block. The block order was pseudorandomized both at the piloting and at the actual cohort stage.

Supplementary Table 2. Number (#) of correct rejection trials (CR), false alarm trials (FA), hit trials (H) and miss trials (M), during sitting and walking, for each one of the 5 pilot participants.

|  | Sitting | | | | Walking | | | |
| --- | --- | --- | --- | --- | --- | --- | --- | --- |
|  | # CR | # FA | # H | # M | # CR | # FA | # H | # M |
| P1 | 29 | 112 | 961 | 84 | 45 | 98 | 963 | 82 |
| P2 | 143 | 35 | 1223 | 8 | 135 | 20 | 1042 | 3 |
| P3 | 49 | 13 | 418 | 0 | 75 | 49 | 835 | 1 |
| P4 | 109 | 46 | 1043 | 2 | 132 | 23 | 1045 | 0 |
| P5 | 139 | 13 | 1041 | 4 | 132 | 20 | 1030 | 15 |

## **Experimental Data**

Number of trials per condition

Supplementary Table 3 shows the mean and standard deviation of the number of trials for each of the 4 behavioral conditions of the Go-NoGo task and for each of the two (2) motor load conditions, across the 26 participants of the entire experimental cohort.

Supplementary Table 3. Number of trials (mean ± standard deviation), during sitting and walking, for each of the 4 behavioral task conditions (CR, FA, H, M).

|  | # CR | # FA | # H | # M |
| --- | --- | --- | --- | --- |
| Sitting | 98 ± 36 | 59 ± 23 | 995 ± 149 | 72 ± 89 |
| Walking | 83 ± 38 | 42 ± 23 | 799 ± 279 | 37 ± 52 |

**Dynamic Time Warping (DTW) Description**

DTW is an algorithm for measuring the similarity between time signals. In the case of one-dimensional signals, if X_m=1,2,..,M_ the reference signal and Y_n=1,2,..,N_ the test signal, then DTW finds a sequence {ix, iy}

of indices (called warping path), such that X(ix) and Y(iy) have the smallest possible distance. The ix and iy are monotonically increasing indices to the elements of signals X, Y respectively, such that elements of these signals can be indexed repeatedly as many times as necessary to expand appropriate portions of the signals and thus achieve the optimal match. This concept can be generalized to multidimensional signals too, like the 3D gait cycle trajectories which are of interest here. The minimal distance between the reference and the test signals (gait trajectories here) is given by the equation below:

$distance=\sum_{\begin{aligned} m\in ix \\ n\in iy \end{aligned}} d_{mn}(X, Y) (1)$

**References**

De Sanctis P, Butler JS, Malcolm BR, Foxe JJ. 2014. Recalibration of inhibitory control systems during walking-related dual-task interference: a mobile brain-body imaging (MOBI) study. Neuroimage 94:55-64.

Rosenthal R. 1991. Meta-analytic procedures for social research, Rev. ed. Meta-analytic procedures for social research, Rev. ed.: Sage Publications, Inc. p. x, 155-x, 155.
